# Supplementary material for: Content-rich biological network constructed by mining PubMed abstracts
Source: BMC Bioinformatics. 2004 Oct 8;5:147. doi: 10.1186/1471-2105-5-147 (PMC528731; doi:10.1186/1471-2105-5-147)
Supplement: Additional File 2 — The original results of the above study (non-essential files are deleted to keep the file size under the limit set by BMC bioinformatics). [file 1471-2105-5-147-S2.bz2 › chilibotAdditionalFile2/dip50/218ID9694899E726/html/IL6R_IL6.html]

 


 **IL6R** and **IL6** 
  
Found 377 abstracts in PubMed, retrieved 50.  
 

 What does Google say? 
 PDF only 
| .edu only 

---

**Interactive relationship** (e.g. stimulation, inhibition, etc)

**Stimulatory relationship**- The mRNAs of both receptors of IL 6  [ **IL6** ] , the soluble  **IL6R**  and gp130, were increased subsequently to IL 6  [ **IL6** ]  mRNA.  Ref: 12733076 Pflugers Arch< MedlineTA>Pflugers Arch,
- BACKGROUND It has recently been shown that soluble interleukin 6  [ **IL6** ]  receptor  [ **IL6R** ]  sIL 6R alone or complexed with interleukin IL 6  [ **IL6** ] , besides their regulatory role in a wide variety of both normal and abnormal biologic reactions mediated by IL 6  [ **IL6** ] , could be an effective stimulator of the cell function.  Ref: 12467526 Mediators Inflamm, 2002
**Neutral relationship**- The human interleukin 6  [ **IL6** ]  receptor  [ **IL6R** ]   **IL6R**  is responsible for signal transduction of  **IL6**  that might have associations with immune diseases and various infectious diseases.  Ref: 12655571 Hum Mutat, 2003
- In contrast, interleukin 6  [ **IL6** ]  was only active at very high concentrations whereas the fusion protein of interleukin 6  [ **IL6** ]  coupled to the soluble interleukin 6  [ **IL6** ]  receptor  [ **IL6R** ]  Hyper IL 6  [ **IL6** ]  exhibited high neurotrophic activity at the same concentrations as ciliary neurotrophic factor.  Ref: 12150983 Biochem Biophys Res Commun, 2002
- Protein kinase C delta associates with the interleukin 6  [ **IL6** ]  receptor  [ **IL6R** ]  subunit glycoprotein gp 130 via Stat3 and enhances Stat3 gp130 interaction.  Ref: 12361954 J Biol Chem, 2002
- Proteolysis of interleukin 6  [ **IL6** ]  receptor  [ **IL6R** ]  IL 6R by Porphyromonas gingivalis cysteine proteinases gingipains inhibits interleukin 6  [ **IL6** ]  mediated cell activation.  Ref: 12079407 Microb Pathog, 2002

**Non-interactive relationship** (e.g. studied together, co-existance, homology, etc.)

- Release of neutrophil IL 6  [ **IL6** ]  receptor alpha IL 6Ralpha, CD126  [ **IL6R** ]  in its soluble form is a key step of IL 6  [ **IL6** ]  transsignaling its physiological inducers are poorly known.  Ref: 12355450 Eur J Immunol, 2002
- METHODS The activation of  **IL6** ,  **IL6R**  and gp130 messenger ribonucleic acid mRNA and protein was studied via reverse transcription polymerase chain reaction RT PCR and immunohistology in donor hearts n = 6 and compared with patients undergoing evaluation of ventricular arrhythmias control, n = 9 or with advanced heart failure n = 20.  Ref: 11985915 J Am Coll Cardiol, 2002
- The interleukin 6  [ **IL6** ]  interleukin 6  [ **IL6** ]  receptor  [ **IL6R** ]  system is activated in donor hearts.  Ref: 11985915 J Am Coll Cardiol, 2002
- Co expression of interleukin 6  [ **IL6** ]  IL 6  [ **IL6** ]  and interleukin 6  [ **IL6** ]  receptor  [ **IL6R** ]  IL 6R in thyroid nodules is associated with co expression of CD30 ligand CD30 receptor.  Ref: 12553555 J Endocrinol Invest, 2002
- Here we report the cloning of a novel type I cytokine receptor, gp130 like monocyte receptor GLM R, with homology to the interleukin 6  [ **IL6** ]  receptor  [ **IL6R** ]  signal transducing chain, gp130, and granulocyte colony stimulating factor receptor.  Ref: 11877449 J Biol Chem, 2002
- RESULTS Messenger RNA of  **IL6** ,  **IL6R**  and gp130 was strongly expressed in all chambers of donor hearts.  Ref: 11985915 J Am Coll Cardiol, 2002
- To address the question whether the neurotrophic effect of interleukin 6  [ **IL6** ]  type cytokines requires soluble cytokine receptors we used fusion proteins of interleukin 6  [ **IL6** ]  coupled to the soluble interleukin 6  [ **IL6** ]  receptor  [ **IL6R** ]  and ciliary neurotrophic factor coupled to the soluble ciliary neurotrophic factor receptor.  Ref: 12150983 Biochem Biophys Res Commun, 2002
- Furthermore, clonal cells expanded from single Lin CD34 CD38 Flk2 Flt3 cells in the culture with Flk2 Flt3 ligand, stem cell factor, thrombopoietin, and a complex of interleukin 6  [ **IL6** ]  soluble interleukin 6  [ **IL6** ]  receptor  [ **IL6R** ]  were individually transplanted into NOD SCID mice.  Ref: 12406096 Br J Haematol, 2002
- These data indicate that interleukin 6  [ **IL6** ]  receptor  [ **IL6R** ]  expression is very low on hippocampal neurons.  Ref: 12150983 Biochem Biophys Res Commun, 2002
- This study investigated the effect of ethinylestradiol EE2 containing oral contraceptives on mineral and bone metabolism and on serum soluble interleukin 6  [ **IL6** ]  receptor  [ **IL6R** ]  sIL 6R during the menstrual cycle.  Ref: 11907703 Calcif Tissue Int, 2002
- Data on the expression of interleukin 6  [ **IL6** ]  IL 6  [ **IL6** ]  interleukin 6  [ **IL6** ]  receptor  [ **IL6R** ]  IL 6R in thyroid nodules is scarce.  Ref: 12553555 J Endocrinol Invest, 2002
- Effect of soluble interleukin 6  [ **IL6** ]  receptor alpha  [ **IL6R** ]  and interleukin 6  [ **IL6** ]  secreted by polymorphonuclear leukocytes on tumor necrosis factor alpha expression and its production by peripheral blood mononuclear cells.  Ref: 12467526 Mediators Inflamm, 2002
- Soluble interleukin 6  [ **IL6** ]  receptor  [ **IL6R** ]  sIL 6R mediates colonic tumor cell adherence to the vascular endothelium a mechanism for metastatic initiation ? < ArticleTitle>.  Ref: 12384057 J Surg Res, 2002
- Cellular cholesterol depletion triggers shedding of the human interleukin 6  [ **IL6** ]  receptor  [ **IL6R** ]  by ADAM10 and ADAM17 TACE.  Ref: 12832423 J Biol Chem< MedlineTA>J Biol Chem, 2002
- in addition, macrophage derived chemokine MDC, thymus and activation regulated chemokine TARC, soluble interleukin 6  [ **IL6** ]  receptor  [ **IL6R** ]  sIL 6R, and soluble tumor necrosis factor receptor I sTNF RI were induced by TNF alpha treatment.  Ref: 11923841 Nat Biotechnol, 2002
- Structure of the extracellular domains of the human interleukin 6  [ **IL6** ]  receptor alpha chain  [ **IL6R** ] .  Ref: 12461182 Proc Natl Acad Sci U S A, 2002
- In addition, the expression of ciliary neurotrophic factor receptor CNTFR and leukemia inhibitory factor receptor LIFR mRNAs, but not of interleukin 6  [ **IL6** ]  receptor  [ **IL6R** ]  mRNA, was up regulated after systemic lipopolysaccharide treatment.  Ref: 12127095 Neuroscience, 2002
- Right ventricles of failing hearts showed  **IL6** ,  **IL6R**  and gp130 mRNA levels comparable with those found in donor hearts.  Ref: 11985915 J Am Coll Cardiol, 2002
- Identification of novel SNPs in the interleukin 6  [ **IL6** ]  receptor  [ **IL6R** ]  gene  **IL6R** .  Ref: 12655571 Hum Mutat, 2003
- The interleukin 6  [ **IL6** ]  receptor  [ **IL6R** ]  can serve as an alpha receptor for CTNF.  Ref: 12643274 J Biol Chem, 2003
- Hybridoma that produces rat anti mouse interleukin 6  [ **IL6** ]  receptor  [ **IL6R** ]  IL 6R antibody, MR16 1, was established by the fusion of mouse P3U1 myeloma cells and spleen cells from mouse soluble IL 6R sIL 6R immunized Wistar rat.  Ref: 12413742 Immunol Lett, 2002
- In vivo activation of the interleukin 6  [ **IL6** ]  receptor  [ **IL6R** ]  gp130 signaling pathway in pituitary corticotropes of lipopolysaccharide treated rats.  Ref: 12624539 Neuroendocrinology, 2003
- Analysis of myeloma cell derived plasma membrane fractions shows that caveolin 1 is co localized with interleukin 6  [ **IL6** ]  receptor  [ **IL6R** ]  signal transducing chain gp130 and with insulin like growth factor I receptor.  Ref: 12482878 J Biol Chem, 2003
- Here we show that the interleukin 6  [ **IL6** ]  receptor  [ **IL6R** ]  IL 6R is rapidly and efficiently inactivated by gingipains, the arginine HRgpA and RgpB and lysine Kgp specific cysteine proteinases from Porphyromonas gingivalis.  Ref: 12079407 Microb Pathog, 2002
- Clinical significance of interleukin 6  [ **IL6** ]  and interleukin 6  [ **IL6** ]  receptor  [ **IL6R** ]  expressions in oral squamous cell carcinoma.  Ref: 12211049 Head Neck, 2002
- Influences of anti mouse interleukin 6  [ **IL6** ]  receptor  [ **IL6R** ]  antibody on immune responses in mice.  Ref: 12413741 Immunol Lett, 2002
- 2 mediators and receptors of immunity, i.e. neopterin, soluble interleukin 2 receptor sIL 2R, soluble interleukin 6  [ **IL6** ]  receptor  [ **IL6R** ]  sIL 6R, soluble tumor necrosis factor receptor sTNF RII.  Ref: 12708230 Int J Hyg Environ Health, 2003
- Transcription levels of tumor necrosis factor receptor TNFR p55 and p75, interleukin 6  [ **IL6** ]  receptor alpha  [ **IL6R** ]  IL 6Ralpha and transforming growth factor beta receptor 1 TGFbetaR1 were quantified in cells exposed to an intensity of 0.1 or 0.8 mT for periods ranging from 30 min to 72 h.  Ref: 12111754 Bioelectromagnetics, 2002
- Soluble interleukin 6  [ **IL6** ]  receptor  [ **IL6R** ]  enhanced by oncostatin M induces major changes in gene expression profile of human hepatoma cells.  Ref: 12008038 Immunol Lett, 2002
- In this report, serum levels of IL 6  [ **IL6** ]  and a soluble form of the interleukin 6  [ **IL6** ]  receptor  [ **IL6R** ]  sIL 6R were evaluated in patients with hepatocellular carcinoma.  Ref: 12095927 Ann N Y Acad Sci, 2002
- 0.001, soluble Interleukin 6  [ **IL6** ]  receptor  [ **IL6R** ]  sIL 6r.  Ref: 12067279 Clin Lab Haematol, 2002
- MEASUREMENTS AND MAIN RESULTS In endotoxin treated rats, there was significant inhibition of interleukin 6  [ **IL6** ]  activation of janus kinase 1, gp 130, the interleukin 6  [ **IL6** ]  receptor  [ **IL6R** ] , STAT1, and STAT3.  Ref: 11902263 Crit Care Med, 2002
- Characterization of anti mouse interleukin 6  [ **IL6** ]  receptor  [ **IL6R** ]  antibody.  Ref: 12413742 Immunol Lett, 2002
- Since hydroxamic acid based metalloprotease inhibitors prevent shedding of both TNFR1 and the interleukin 6  [ **IL6** ]  receptor  [ **IL6R** ]  IL 6Ra, we hypothesized that ARTS 1 might also regulate shedding of IL 6Ra, a member of the type I cytokine receptor superfamily that is structurally different from TNFR1.  Ref: 12748171 J Biol Chem< MedlineTA>J Biol Chem, 2002
- An aminopeptidase, ARTS 1, is required for interleukin 6  [ **IL6** ]  receptor  [ **IL6R** ]  shedding.  Ref: 12748171 J Biol Chem< MedlineTA>J Biol Chem, 2002
- Stability of interleukin 6  [ **IL6** ] , soluble interleukin 6  [ **IL6** ]  receptor  [ **IL6R** ] , interleukin 10 and CC16 in human serum.  Ref: 12393169 Cytokine, 2002
- Biological activity of a novel nonpeptide antagonist to the interleukin 6  [ **IL6** ]  receptor  [ **IL6R** ]  20S, 21 epoxy resibufogenin 3 formate.  Ref: 12235239 J Pharmacol Exp Ther, 2002
- Interleukin 6  [ **IL6** ]  receptor  [ **IL6R** ]  expression and localization after transient global ischemia in gerbil hippocampus.  Ref: 12676341 Neurosci Lett, 2003
- Interleukin 6  [ **IL6** ] , soluble interleukin 2 receptor and soluble interleukin 6  [ **IL6** ]  receptor  [ **IL6R** ]  in the sera of patients with different histological patterns of rheumatoid synovitis.  Ref: 12673891 Clin Exp Rheumatol< MedlineTA>Clin Exp Rheumatol, 2003
- Relationship between circulating serum soluble interleukin 6  [ **IL6** ]  receptor  [ **IL6R** ]  and the angiogenic cytokines basic fibroblast growth factor and vascular endothelial growth factor in multiple myeloma.  Ref: 12574959 Ann Hematol, 2003
- Aim To measure serum concentrations of the soluble interleukin 2 receptor sIL2R, soluble interleukin 4 receptor sIL4R, soluble interleukin 6  [ **IL6** ]  receptor  [ **IL6R** ]  sIL6R, and soluble tumor necrosis factor alpha receptor I and soluble tumor necrosis factor alpha receptor II, during the perinatal and early neonatal period, in order to evaluate their role in activation of immune response in labor and the first days postpartum.  Ref: 12857603 Mediators Inflamm, 2003
- Increased soluble interleukin 6  [ **IL6** ]  receptor  [ **IL6R** ]  in vitreous fluid of proliferative vitreoretinopathy.  Ref: 12789531 Curr Eye Res, 2003
- Prostaglandin E2 mediates growth arrest in NFS 60 cells by down regulating interleukin 6  [ **IL6** ]  receptor  [ **IL6R** ]  expression.  Ref: 12429018 Biochem J, 2003
- We studied the production of GRO alpha by human umbilical vein endothelial cells in response to the stimulation with soluble form of interleukin 6  [ **IL6** ]  receptor alpha  [ **IL6R** ] .  Ref: 12008100 Life Sci, 2002
- Differential effects of interleukin 6  [ **IL6** ]  receptor  [ **IL6R** ]  activation on intracellular signaling and bone resorption by isolated rat osteoclasts.  Ref: 12065229 J Endocrinol, 2002
- Part II Significance of anti interleukin 6  [ **IL6** ]  and anti soluble interleukin 6  [ **IL6** ]  receptor alpha  [ **IL6R** ]  antibodies in a standardized murine contact burn model.  Ref: 12771624 Crit Care Med, 2003
- Oral contraceptives moderately effect bone resorption markers and serum soluble interleukin 6  [ **IL6** ]  receptor  [ **IL6R** ]  concentrations.  Ref: 11907703 Calcif Tissue Int, 2002
- Cerebrospinal fluid and serum protein levels of tumour necrosis factor alpha TNF alpha interleukin 6  [ **IL6** ]  IL 6  [ **IL6** ]  and soluble interleukin 6  [ **IL6** ]  receptor  [ **IL6R** ]  sIL 6R gp80 in multiple sclerosis patients.  Ref: 12445803 Cytokine, 2002
- Replacing the TfR stalk by the corresponding sequences from tumor necrosis factor alpha or interleukin 6  [ **IL6** ]  receptor  [ **IL6R** ]  also led to TfR ectodomain shedding.  Ref: 12586332 FEBS Lett, 2003
- We have determined the percentage of alphabeta and gammadelta T cells by flow cytometry as well as serum interleukin 6  [ **IL6** ]  IL 6  [ **IL6** ]  and soluble interleukin 6  [ **IL6** ]  receptor  [ **IL6R** ]  sIL 6R levels by enzyme linked immunosorbent assay in kidney allograft recipients with acute, chronic or stable graft evolution.  Ref: 12828564 Scand J Immunol< MedlineTA>Scand J Immunol, 2003
- Using the recently engineered designer cytokine IC7, which induces an active heterodimer of the LIFR and gp130 after binding to the IL 6R, and several receptor chimeras of the LIFR and the interleukin 6  [ **IL6** ]  receptor  [ **IL6R** ]  IL 6R carrying the CBM of the IL 6R in place of the COOH terminal LIFR CBM, we could assign individual receptor subdomains to individual binding sites of the ligand.  Ref: 12731865 Biochemistry, 2003
- The effects of interleukin 6  [ **IL6** ]  on key intermediates in early steps of the interleukin 6  [ **IL6** ]  signaling pathway, including janus kinase 1, gp 130, the interleukin 6  [ **IL6** ]  receptor  [ **IL6R** ] , STAT1, and STAT3, were examined in both saline and endotoxin treated rats.  Ref: 11902263 Crit Care Med, 2002
- Polymorphisms of the gene encoding the inflammatory cytokine interleukin 6  [ **IL6** ]  determine the magnitude of the increase in soluble interleukin 6  [ **IL6** ]  receptor  [ **IL6R** ]  levels in Alzheimer s disease Results of a pilot study.  Ref: 12664314 Eur Arch Psychiatry Clin Neurosci, 2003
- Gp130 activation by soluble interleukin 6  [ **IL6** ]  receptor  [ **IL6R** ]  interleukin 6  [ **IL6** ]  enhances osteoblastic differentiation of human bone marrow derived mesenchymal stem cells.  Ref: 12372336 Exp Cell Res, 2002
- Its action requires expression of the specific  **IL6**  receptor  **IL6R** , and the common signal transducer of the  **IL6**  family glycoprotein 130 gp130 in the donor heart.  Ref: 11985915 J Am Coll Cardiol, 2002
- A synthetic corticosteroid, dexamethasone regulates generation of soluble form of interleukin 6  [ **IL6** ]  receptor  [ **IL6R** ]  of human lymphocytes, in vitro.  Ref: 12371610 Acta Biol Hung, 2002
